# Efficacy of a silver colloidal gel against selected oral bacteria *in vitro*

**DOI:** 10.12688/f1000research.17707.1

**Published:** 2019-03-07

**Authors:** Phat L. Tran, Keaton Luth, James Wang, Coby Ray, Anselm de Souza, Dilip Mehta, K. W. Moeller, C. D. Moeller, Ted W. Reid

**Affiliations:** 1 Departments of Ophthalmology and Visual Sciences, Texas Tech University Health Sciences Center, Lubbock, Texas, USA; 2 Viridis BioPharma Pvt Ltd, Mumbai, Maharashtra, India; 3 American Biotech Labs LLC, Alpine, Utah, USA; 4 Selenium Ltd, Austin, TX, USA

**Keywords:** Silver, biofilm, dental plaque, dental caries

## Abstract

**Background: **It is necessary to develop new strategies to protect against bacteria such as S *treptococcus mutans*, S *treptococcus sanguis*, and *Streptococcus salivarius*, which contribute to tooth decay and plaque formation. Our current study investigated the efficacy of a colloidal silver gel in inhibiting biofilm formation by these principal oral bacteria *, in vitro*. The aim of this study was to assess the efficacy of a colloidal silver gel formulation for inhibiting bacterial biofilm formation (Ag-gel) by the principal bacteria that cause plaque formation and tooth decay.

**Methods:** The effect of Ag-gel on viability of *S. mutans*, *S. sanguis*, *and S. salivarius* was assessed by quantifying their colony forming units (CFU) in presence or absence of the test gel. The effect of this formulation on biofilm-forming ability of these bacteria was studied through scanning electron microscopy.

**Results: **Using the CFU assays, over 6 logs of inhibition (100%) were found for *S. mutans*, *S. sanguis*, and *S. salivarius* for the Ag-gel-treated bacteria when compared with the control gel. In addition, the Ag-gel also inhibited biofilm formation by these three bacteria mixed together. These results were confirmed by scanning electron microscopy.

**Conclusions:** The Ag-gel was effective in preventing biofilm formation by *S. mutans, S. sanguis, and S. salivarius*. This Ag-gel should be tested for the ability to block plaque formation in the mouth, through its use as a tooth paste.

## Introduction

Problems associated with maintenance of oral health are faced by many people throughout the world, irrespective of their age and gender. The most common oral problems amongst all are dental caries, bleeding gums (periodontal diseases) and oral cancers ^1^. Over few decades, the severity and prevalence of dental caries, and oral cancer, which can be a fatal condition, have increased ^2, 3^.

In the US, caries were estimated to be five times as common as asthma and seven times as common as allergic rhinitis ^3^. According to the World Health Organization (WHO), dental caries are caused due to high sugar consumption, which is also linked with being overweight and obesity ^4^. The incidence of periodontal diseases is estimated to be about 20–50% of the global population ^5^. There are two approaches for the management of caries: extraction and preventions. The primary treatment modality for these caries, though very painful, is extraction of carious teeth ^2, 3^. The routine prevention measures for dental caries are to maintain oral hygiene, involving the use of fluoride toothpaste and/or xylitol ^6, 7^. Looking at the currently prevailing painful treatment, there is a need for new products to be developed for the prevention of oral cavities. The pathological organisms responsible for these caries/ periodontal diseases are *Streptococcus mutans*, *Streptococcus sanguis* and *Streptococcus salivarius.*

Since mouth washes and different tinctures have been found to be ineffective against dental biofilm formation ^8^, finding novel products effective against cariogenic microbes like *S. mutans* is important. While Listerine ^®^, has some antimicrobial activity, toothpastes such as Toss-K and Senquel-AD have no activity against four important dental caries pathogens ^9^. Thus, the search continues for more effective agent(s) ^10^. A novel product that is effective against biofilm formation would be an important contribution to chewing sticks, toothpastes or other dental products.

*S. mutans* has the ability to adhere the enamel surface, produce acid metabolites, build glycogen reserves and to synthesize extracellular polysaccharides. Mutans streptococci create acidic environment creating a risk for formation of cavity. During the formation of dental plaque, *S. mutans* adhere to primary colonizers by cell to cell interaction which forms biofilm on the teeth which induces bacterial growth ^11^.

*Streptococcus sanguis* is normally found in the human oral cavity. Due to low cariogenicity, it forms a colony on tooth surface which gets aggregated by other oral bacteria and leads to maturation of dental plaque ^12^. Another organism, *Streptococcus salivarius* belonging to the *salivarius* subspecies is found in oral cavity in humans a few hours after birth and remain there as the predominant inhabitant. All these organisms enhance caries formation and thus the progression of periodontal disease. The aforementioned treatment modalities are unsuccessful in controlling or killing these bacteria and hence in prevention of caries.

Silver has been used since ancient times as antibacterial agent for various pathological elements. During the last century, the antimicrobial action of silver has been investigated ^13^. Colloidal silver is observed to be less toxic than ionic silver and has good compatibility with human cells. Silver was found to be effective in dentine desensitizer and is used as root canal disinfectant ^14^. Silver nanoparticles are also used in dental material depending on the type of material being used. For example, titanium samples are mainly soaked in AgNO _3_ solution for dental implants to avoid bacterial contamination ^15^. The mechanism of action of silver compounds on carious tooth is to inhibit demineralization process and anti-bacterial effect by interfering with bacterial cell membrane, cytoplasmic enzyme and inhibition of DNA replication of bacteria ^14^.

Oral health being a global concern, it is essential to develop strategies to prevent dental caries and plaque formation. This study aimed at investigating the efficacy of a colloidal silver gel in inhibiting biofilm formation *in vitro* by the principal oral bacteria, *Streptococcus mutans*, *Streptococcus sanguis* and *Streptococcus salivarius.*

## Methods

### Bacterial strains, media, and growth conditions

*Streptococcus salivarius* strain ATCC® 13419 ^TM^, *Streptococcus sanguis* ATCC ^®^ 10556 ^TM^, and *Streptococcus mutans* strain ATCC® 35668 ^TM^ were obtained from Remel (Lenexa, KS, USA). *S. salivarius*, *S. sanguis*, and *S. mutans* were routinely grown in Brain Heart Infusion (BHI, #53286, Sigma-Aldrich, St. Louis, MO), at 37°C for 24 h.

### Colloidal silver materials

Colloidal silver in a gel form was obtained from Viridis BioPharma Pvt., Ltd. (Mumbai, India). It was tested by evenly spreading 0.5 g on a 6-mm blank paper disc (BD Diagnostic System, Sparks, US) inoculated with the bacteria listed in the above paragraph. We assessed the bacteria remaining on the disc by the CFU assays below.

### *In vitro* colony forming unit (CFU) assays

A total of three blank sterile (6 mm diameter) cellulose paper discs were placed onto individual LB agar plates. Approximately 1x10 ^3^ CFUs of the test bacteria were inoculated onto each disc. In the biofilm mixture study, approximately 4x10 ^2^ of each bacterium were combined together. Either no gel (untreated), Ag-gel, or placebo gel (Viridis gel without Ag), were placed over the discs inoculated with bacteria. The plates were incubated under micro-aerobic conditions, which were generated by placing the plates inside a gas jar containing an EZ GasPak (Catalog no. 260678, BD, Franklin Lakes, NJ, USA) at 37°C for 24 h. Following incubation, each cellulose disc was analyzed for the remaining viable bacteria by the CFU assay as previously described ^16^. Each piece was carefully removed from the well, rinsed gently with sterile distilled H _2_O, and placed in a microcentrifuge tube containing 1 ml PBS. The tubes were placed in a water bath sonicator for 5 min to loosen the cells within the biofilm and then vigorously vortexed 3 times for 1 min to detach the cells. Suspended cells were serially diluted 10-fold in PBS, and 10-µl aliquots of each dilution were spotted onto BHI plates. The plates were incubated at 37°C for 24 h. In experiments where no bacteria were detected, the remaining 900 µl of undiluted samples were tested. Thus, the equation for back-calculating the bacterial concentration was CFU x dilution factor x 100, with the exception of the 100-µl sample which was calculated as CFU x 10. This means that the smallest number of bacteria that we could detect would be approximately 1 bacterium. All experiments were performed in triplicate.

### Scanning electron microscopy (SEM)

Biofilms formed on discs were prepared for SEM by standard techniques and the experiment was performed as previously described ^16– 18^. *S. salivarius, S. sanguis, S. mutans* or a mixture of *S. salivarius, S. sanguis,* and *S. mutans* biofilms were established on cellulose discs (with or without silver gel, as described above). After 24 h of incubation, each cellulose disc and any adherent bacteria were fixed with 2% (wt/vol) glutaraldehyde in filter-sterilized 0.05 M PBS (pH 7.4) at room temperature for 16 h and then rinsed three times for 15 min each in 0.05 M PBS. The fixed cellulose discs were then dehydrated in successive ethanol-water mixtures with increasing ethanol concentrations (20%, 40%, 60%, 80%, and 95% [vol/vol]) for 15 min each and then twice in absolute ethanol for 15 min. The ethanol-dehydrated samples were then placed in an absolute ethanol bath, which was placed in an EMS 850 critical point drier (Electron Microscopy Sciences, Hatfield, PA). The ethanol was replaced by successive additions of liquid carbon dioxide. Once the liquid CO _2_ had replaced the ethanol, the chamber was heated under pressure to reach the critical evaporation point of carbon dioxide. The chamber was then slowly vented of gaseous CO _2_ and the dry samples removed. The dried samples were affixed to aluminum mounts with double-sided carbon adhesive tape and sputter-coated with platinum and palladium to a thickness of 18 nm. Observations were performed at 5 to 7 kV with a scanning electron microscope (Hitachi S-570; Japan). Five fields of view at 5,000X-10,000X magnification were taken at randomly chosen areas from the optic surface of each sample. A biofilm-positive field was defined as being occupied by biofilm over at least half of the visible area.

### Statistical analysis

The results of the CFU assays were analyzed with Prism ^®^ version 4.03 (GraphPad Software, San Diego, US) with 95% confidence intervals (CIs) of the difference. Comparisons of the *in vitro* biofilms formed on the cellulose discs with either Ag-gel dressings or Ag-free ones were analyzed by a two-tailed unpaired t-test to determine significant differences. All experiments were done in triplicate. The significance limit was P<0.05.

## Results

### Effect of Ag-gel on bacterial biofilm formation for *in vitro* CFU studies

**Figure 1.**
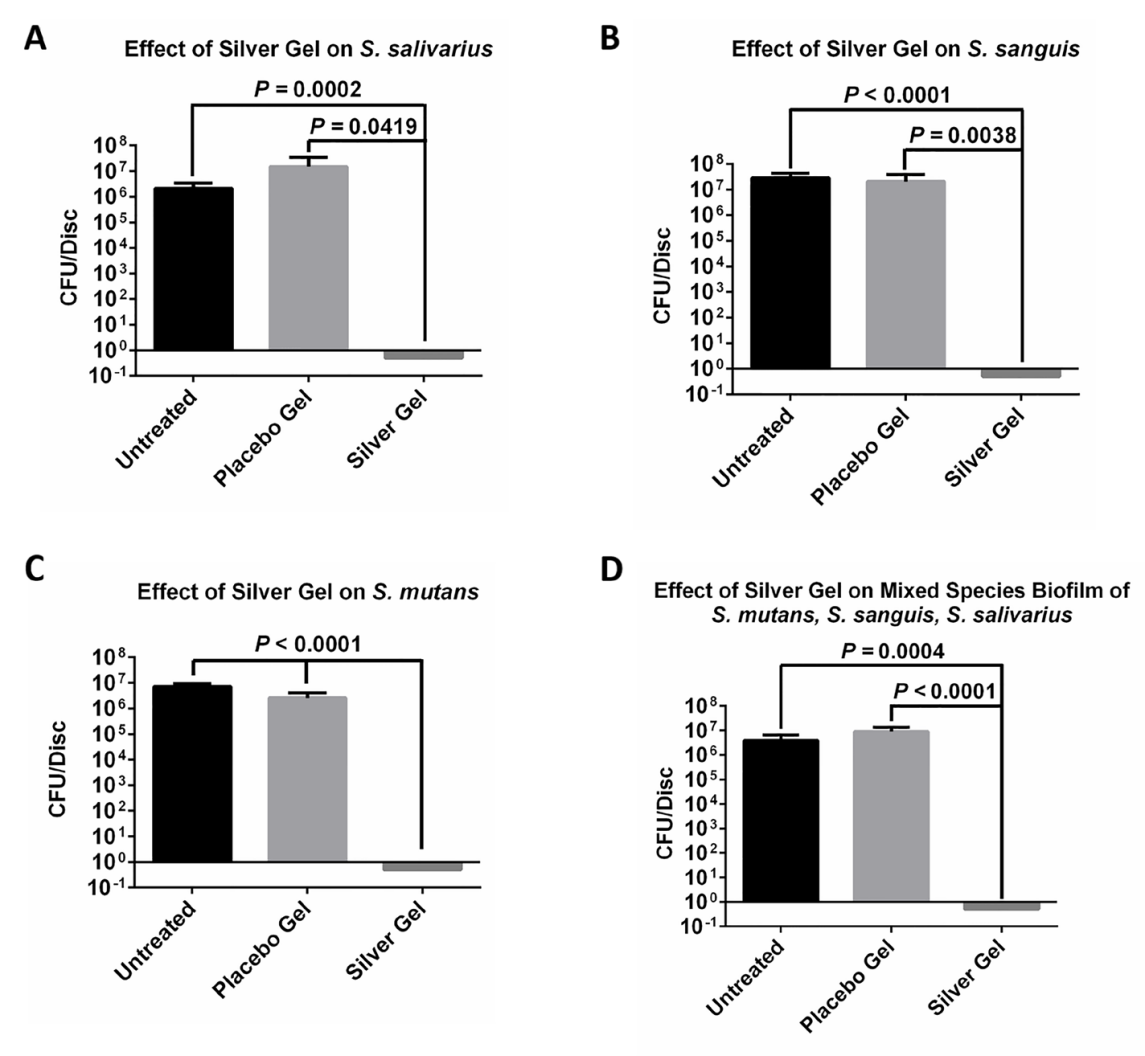
Colony forming unit analysis of biofilm formation. ( **A**) *S. salivarius*, ( **B**) *S. sanguis*, ( **C**) *S. mutans* or ( **D**) all three on untreated discs, discs treated with placebo gel or discs treated with colloidal silver gel.

The results for the 24-h micro-aerobic *in vitro* studies using *S. salivarius*, *S. sanguis*, and *S. mutans* isolates, as well as the mixture of all three, are illustrated in Figure 1. As seen in Figure 1A–D, the cellulose discs that had no treatment, and those that were treated with the placebo gel, showed over 6 logs of bacterial growth in each case. However, the silver containing gel showed 100% inhibition (over 6 log of killing) in all cases with *S. salivarius*, *S. sanguis*, *S. mutans*, or the combination of all three strains, when compared with the control, as showed in Figure 1A–D. Raw data are available on OSF ^19^.

### Effect of Ag-gel dressing on bacterial biofilm formation for *in vitro* SEM studies

**Figure 2.**
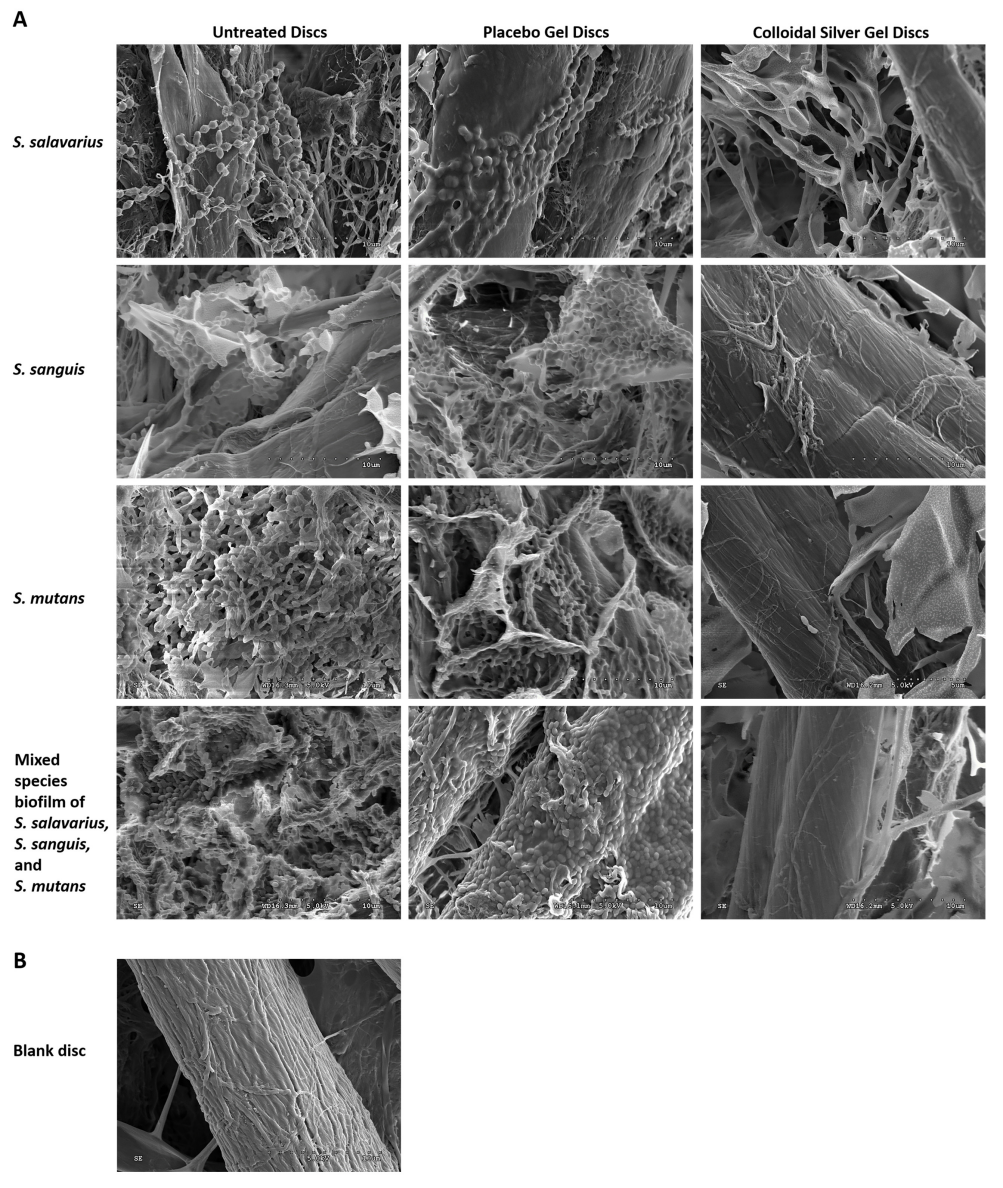
Scanning electron microscopy (SEM) imaging. ( **A**) SEM analysis of *S. salivarius, S. sanguis or S. mutans* biofilm formation on untreated discs, discs treated with placebo gel or discs treated with colloidal silver gel. ( **B**) SEM of a blank disc.

To confirm results of CFU assay described in previous section, the biofilm formation of *S. salivarius*, *S. sanguis*, *S. mutans* or the mixture of all three strains was studied on cellulose discs by SEM. *S. salivarius*, *S. sanguis*, *S. mutans* and the mixture of all three strains, were inoculated onto the discs in the same manner as for the CFU biofilm assay. Untreated discs were coated with placebo gel only. As above the discs were incubated for 24 h under micro-aerobic conditions at 37°C. As seen in Figure 2, *S. salivarius*, *S. sanguis*, *S. mutans* and the mixture of all three strains, formed typical biofilms characterized by the presence of micro-colonies on the cellulose discs receiving no treatment, or treated with the placebo gel. However, no bacteria were seen on the cellulose discs treated with colloidal silver gel. These results confirm those obtained with the CFU assay. Raw SEM images are available on OSF ^19^.

## Discussion

There are over 700 different species that contribute to the formation of dental biofilm (plaque) ^9– 11^. Among these species, *Streptococcus mutans*, *Streptococcus salavarius*, and *Streptococcus sanguinis* are the main members of this plaque ^20– 22^. These bacteria are also considered to be the primary etiologic agents of human dental caries ^23– 26^. It is the interaction of *S. mutans* with other streptococci that is thought to be important in dental plaque formation. ^27^. *S. mutans* can cause cariogenicigty by the production of glucosyltransferase enzymes that allows glucose from sucrose to be used for the synthesis of glucan, and have also been implicated in heart problems ^28, 29^. *S. sanguinis* is an another common organism in dental plaque, which can colonize dental cavities. An additional problem is that this organism is also often found in the bloodstream. This allows it to attach to heart valves, causing bacterial endocarditis ^22^. Thus, *S. sanguinis* is a key agent in infective endocarditis ^21^.

Since *S. salivarius* is also part of the normal human flora, it can contaminate sterile body fluid. Thus, therapeutic interventions that disrupt the cells protecting the blood vessels can allow it to enter the blood stream and cause problems in areas such as the meninges and the cerebrospinal fluid. ^30– 33^. This results in a variety of infections such as meningitis and bacteraemia along with many other bacterial problems. ^27, 30, 34– 39^. Thus, these three bacterial species can not only work together to form plaque on teeth, but can play a major role in other medical problems in the body.

The objective of the current study was to evaluate the test Ag-gel for its efficacy to either control or annihilate the growth of these 3 organisms. The results present quantitative data of the antimicrobial effect on *S. salivarius*, *S. sanguis*, *or S. mutans* bacteria, of Ag-gel, layered on a cellulose disc which was inoculated with these bacteria or a combination of all three bacterial strains. The CFU assay results of *in vitro* studies using Ag-gel treated dressings showed over 6 log of killing (100%) for *S. salivarius*, *S. sanguis*, *or S. mutans* as compared with a control gel dressing containing no Ag-gel. Since biofilms adhere strongly to surfaces, the experiments were also studied by SEM. These SEM studies confirmed the CFU results with *S. salivarius*, *S. sanguis*, *or S. mutans* biofilms, in the presence of Ag-gel or placebo gel. As seen in Figure 2, mature biofilms formed in the presence of the placebo gel, but none in the presence of the Ag-gel.

The mixture of these three bacteria was also studied, since it is proposed that the combination of bacteria is more resistant to growth inhibition than the individual bacteria. Similar results to the individual bacteria, (>6-log kill rate, 100%) were obtained with the combination of the three bacteria by both the CFU assay and by SEM studies.

## Conclusion

An Ag-gel was found to be capable of over 6 log (100%) inhibition of *S. salivarius, S. sanguis, or S. mutans* bacteria, or a mixture of all three bacteria forming biofilms on cellulose discs by CFU studies. These results were confirmed by SEM studies of biofilm formation by *S. salivarius, S. sanguis,* or *S. mutans* or a mixture of all three bacteria, where the Ag-gel dressing showed total inhibition of biofilm formation on cellulose discs. These results indicate that use of a colloidal silver gel is an effective way to inhibit the formation of biofilms by the most common bacteria implicated in oral plaque formation, and this gel stands good potential to be developed into an effective commercial dentifrice product.

## Data availability

Raw data for this study are available on OSF. DOI: https://doi.org/10.17605/OSF.IO/AJNYU ^19^.

Data are available under the terms of the Creative Commons Zero "No rights reserved" data waiver (CC0 1.0 Public domain dedication).
