# Supplementary material for: Efficacy of a silver colloidal gel against selected oral bacteria in vitro
Source: F1000Res. 2019 Mar 7;8:267. [Version 1] doi: 10.12688/f1000research.17707.1 (PMC6468711; doi:10.12688/f1000research.17707.1)
Supplement: Peer review history

# Peer review: Reviewer response for version 1

**DOI:** 10.5256/f1000research.19360.r47334

**Published:** 2019-05-15

**Authors:** C S Srinandan

**Affiliations:** 1 Biofilm Biology Lab, Centre for Research on Infectious Diseases, School of Chemical and Biotechnology, SASTRA Deemed University, Thanjavur, India

This study by Tran *et al*., is important, but preliminary report that shows colloidal silver to be effective against three Streptococcus species. Following are some of the concerns I have regarding this manuscript.

1. The authors state that “the Ag-gel should be tested for the ability to block plaque formation in the mouth, through its use as a toothpaste”. Let’s consider the following: (a) Oral microbiome is very rich and its composition is important for health status of an individual and (b) Literature says colloidal silver is toxic to the host cells.  

Considering (a) and (b), using the Ag-gel as a toothpaste may not be a good idea as it is not specific towards the pathogens. Thus, I recommend the authors to measure the cost-benefit ratio and mention about the conditions or stages of oral disease at which it could possibly be used as a toothpaste.

1. Why only one concentration of 0.5g of colloidal silver used for the studies?
2. Please provide the details for the figures in the legends, like (i) what is the sample size, (ii) what do the error bars indicate, and (iii) which statistical test was performed on the data.
3. I fail to understand the biological relevance of negative data of CFU in Fig. 1.
4. What happens to biofilm formation at lower concentrations of silver?
5. As the previous reviewers commented, eradication of biofilms should also be tested. Therefore, this manuscript should accompany the Minimum Biofilm Inhibitory Concentration (MBIC) and Minimum Biofilm Eradication Concentration (MBEC) of colloidal silver.
6. Determination of interaction of colloidal silver with antibiotics is also an important test that has to be performed.

I have read this submission. I believe that I have an appropriate level of expertise to confirm that it is of an acceptable scientific standard, however I have significant reservations, as outlined above.

# Peer review: Reviewer response for version 1

**DOI:** 10.5256/f1000research.19360.r45453

**Published:** 2019-04-16

**Authors:** Mohan Kulkarni

**Affiliations:** 1 Department of Chemistry, Biochemistry Division, Savitribai Phule Pune University, Pune, India

Caries has been the one of the most prevalent dental conditions which needs effective treatment. Products currently in use such as mouthwashes recommended for oral hygiene are not very effective against dental biofilm formation.

 This study evaluates the efficacy of colloidal silver gel formulations in inhibiting bacterial biofilm formation by *S. mutans, S. sanguis, and S. salivarius* which cause plaque formation. The choice of colloidal silver over ionic silver in view of its lower toxicity is well justified and so also the choice of bacteria for evaluating the efficacy of colloidal silver.

 The effectiveness of colloidal silver has been demonstrated by quantifying colony forming units (CFU). Appropriate controls and statistical analyses have been provided to support the results. The results are further supported by scanning electron microscopy. The results show that no biofilms are formed in the presence of colloidal silver gel but do form in the control experiments carried out with placebo. 

 Since the objective of the work is to develop a treatment for the prevention of caries, it would be desirable to optimize the concentration of colloidal silver in the gel in future. In addition to prevention of biofilm formation if colloidal silver is shown to be useful in disrupting biofilms already formed, it would enhance the scope of treatment.

 I recommend the indexing of the paper “Efficacy of a silver colloidal gel against selected oral bacteria in vitro”.

I have read this submission. I believe that I have an appropriate level of expertise to confirm that it is of an acceptable scientific standard.

# Peer review: Reviewer response for version 1

**DOI:** 10.5256/f1000research.19360.r45449

**Published:** 2019-03-11

**Authors:** Vijay Kothari

**Affiliations:** 1 Institute of Science, Nirma University, Ahmedabad, India

This study has evaluated antibacterial efficacy of a colloidal silver gel against three important oral bacteria. In context of the currently available dentifrice products not being full effective in preventing the widespread problem of dental caries, need and research for new more effective dentifrices is justified. Their idea of assaying the test gel not only against individual bacteria, but also against the mixed-species is a logical one, as that represents real-life situations a bit more closely. In the recent past, maintenance of good oral health has been shown to be important for good cardiovascular health, healthy pregnancy, etc. 

 They may consider re-framing the last sentence of the third paragraph in 'Introduction'. They may also check for correct placement of all references; For e.g. references 2-3 have been cited in first paragraph in an oral health context, but their titles seem to be more relevant to wound management.

 While, I do not have any reservations in recommending this article in its present form for indexing, I suggest for following additional experiments for future studies by these authors:

1. Effect of this gel on pre-formed biofilms of oral bacteria should be checked on two different parameters i.e. whether this gel can kill bacteria in biofilm, and whether it can eradicate the pre-formed biofilm with/without killing the bacteria in it. 
2. Results of current study indicate their gel to possess a bactericidal action. They may consider extending the incubation till 48-72 hours after transferring the gel-exposed bacteria onto fresh gel-free media, to confirm the true bactericidal effect, and absence of post-antibiotic effect (PAE). 
3. They may consider determination of MIC, MBC, and time required to kill. The latter is important as typical dentifrice products like mouthwashes/toothpaste get few seconds/minutes to exert their effect in the mouth. 
4. It will be interesting to investigate whether at sub-inhibitory concentrations their product can reduce lactic acid production by the Streptococci, which is a major virulence factor involved in demineralization of teeth. Whether sub-inhibitory concentrations can inhibit quorum-sensing in these bacteria?
5. In the current study, cellulose discs were used as surface for biofilm formation. In future studies, they may consider some material similar to teeth-substance (e.g. calcium) as the surface for biofilm formation. 

I have read this submission. I believe that I have an appropriate level of expertise to confirm that it is of an acceptable scientific standard.
